# Continuous perinatal health monitoring and analysis in Europe based on a federated analytic approach: a proof of concept study

**DOI:** 10.1093/eurpub/ckac129.109

**Published:** 2022-10-25

**Authors:** M Philibert, M Amyx, M Durox, J Dimnjakovic, M Thissen, F Estupiñán-Romero, E Berna, J Zeitlin

**Affiliations:** 1 Obstetrical, Perinatal and Pediatric Epidemiology Research Team, Université Paris Cité, Inserm, Paris, France; 2 Divison of Public Health, Croatian Institute of Public Health, Zagreb, Croatia; 3 Department of Epidemiology and Health Monitoring, Robert Koch Institute, Berlin, Germany; 4 Data Sciences for Health Services and Policy, Institute for Health Sciences in Aragon, Zaragoza, Spain

## Abstract

**Background:**

International comparisons of population indicators of maternal and newborn health are valuable for guiding health policy and practice. The Covid-19 pandemic revealed the difficulties of compiling comparable, timely data in Europe. As part of the PHIRI (Population Health Information Research Infrastructure) project, we developed a protocol to facilitate the exchange and analysis of population birth data in Europe.

**Methods:**

The Euro-Peristat network, which includes experts from 31 European countries, developed a common data model and R scripts to facilitate rapid exchange of anonymised aggregate tables (https://zenodo.org/record/5148032#.YmlUttpBxPY). These tables were used to compile comparable perinatal health indicators from routine population-based sources for the years 2015 to 2020. We assessed the feasibility of this approach and the availability, quality and comparability of the data.

**Results:**

Building on previous Euro-Peristat recommendations and a structured consensus process, the network defined a common data model including 22 variables for the testing phase. 17 additional variables were considered important and feasible for a second phase. 25 countries created patient-level data files. Most countries had 20 or more of the data items, whereas 1 had 18, 3 had 16 and 2 had 15 variables. Limiting factors included not having all data in a single database, most often the case for neonatal and infant mortality or vital statistics versus healthcare data, and the diversity or absence of data on socioeconomic status. Setting up the model was time consuming, but once established, running the R scripts was easy and quick (<15 min). The protocol requires the active participation of each country to ensure it is correctly applied.

**Conclusions:**

We illustrated the feasibility of using a common data model with open source scripts to facilitate rapid production of data and analysis on key perinatal health indicators in European countries

